# Concentration sérique en fer au cours de la malnutrition chez l’enfant: cas d’une zone urbaine et rurale en République Démocratique Congo

**DOI:** 10.11604/pamj.2018.31.55.16089

**Published:** 2018-09-25

**Authors:** Aimée Mudekereza Musimwa, Hermann Tamubango Kitoko, Gray Kanteng Wakamb, Stanis Wembonyama Okitotsho, Oscar Luboya Numbi

**Affiliations:** 1 Université de Lubumbashi, Département de Pédiatrie, Lubumbashi, République Démocratique Congo; 2 Centre Médical Georgia, Lubumbashi, République Démocratique Congo

**Keywords:** Malnutrition, fer, enfants, Lubumbashi, Malnutrition, iron, children, Lubumbashi

## Abstract

**Introduction:**

la malnutrition (protéine énergétique et vitaminique) contribue à la survenue d'une anémie. Parallèlement, l'anémie ferriprive est l'un des troubles nutritionnels les plus répandus au monde et particulièrement dans les pays en voie de développement. Ainsi l'objectif de cette étude est de déterminer le taux de fer chez l'enfant malnutri de 6 à 59 mois et les facteurs liés à sa variation.

**Méthodes:**

il s'agit d'une étude transversale menée sur la période allant du 01 juillet 2013 au 31 juillet 2014. Nous avons colligé 180 enfants malnutris dès leur admission dans un centre de prise en charge nutritionnelle. Un autre groupe a été recruté en périphérie de la ville, dans le village Kawama. Une ponction veineuse a été effectuée et le sang prélevé a subi une centrifugation puis une lecture au spectromètre.

**Résultats:**

il ressort que 93,4 % de l'échantillon soit 170 enfants ont présenté une concentration sérique inférieure à la valeur normale. Par contre 6,59% soit 12 enfants ont présenté un taux de fer entre 610-1300 μg/L de fer sérique avec la médiane qui était à 159,5 μg/L. Parmi les 170 enfants qui avaient un taux de fer sérique de <610 μg/L, 79 enfants soit 42,18% étaient âgés de moins de 24 mois dont 3 (1,78%) âgés de moins de 6 mois. A Kawama, 62 enfants soit 36,47% de l'échantillon total avaient un taux de fer <610. Aucun lien statistiquement significatif entre le fer et les facteurs de risque n'a été trouvé sauf pour la variable provenance où le p était significatif (p< 0,05).

**Conclusion:**

la concentration sérique en fer est en baisse chez les enfants malnutris, aiguë comme chronique à leur admission avec une médiane autour de 159,5 μg/L; ceci justifierait les vastes programmes de fortification en fer des différents aliments et/ou l'administration du fer à la première semaine tout en traitant les complications (paludisme, les infections bactériennes et autres infections parasitaires)

## Abstract (en)

**Introduction:**

malnutrition (protein energy malnutrition, vitamin deficiency) contributes to anemia. At the same time, iron-deficiency anemia is one of the most common nutritional disorders in the world, especially in developing countries. This study aims to determine iron concentrations in malnourished children aged 6 -59 months and factors associated with variation in iron levels.

**Methods:**

we conducted a cross-sectional study from 01 July 2013 to 31 July 2014. The study involved 180 malnourished children and the study period began right after their admission in Nutrition Care Center. Another group living in the outskirts of the town, in the village Kawama, was included in the study. Venous puncture was performed, blood collection tubes of each patient were centrifuged and then analyzed by spectroscopy.

**Results:**

one hundred and seventy children (93.4%) had serum concentration lower than the normal value. However, 12 children (6.59%) had serum iron concentration between 610-1300 μg/L with mean concentration of 159.5 μg/L. Out of 170 children with serum iron concentration of <610 μg/L, 79 children (42.18%) were under the age of 24 months, 3 of whom (1.78%) were under the age of 6 months. In Kawama, 62 children (36.47%) had iron concentration <610. There was no statistically significant association between iron and risk factors, except for the provenance variable with P considered significant (p < 0.05).

**Conclusion:**

serum iron concentration were lower in acutely or chronically malnourished children on admission, with a mean iron concentration of approximately 159.5 μg/L; this would justify the vast iron fortification programs of different foods and/or the administration of iron in the first week while treating complications (malaria, bacterial infections and other parasitic infections).

## Introduction

La malnutrition est l'un des problèmes de santé publique affectant le plus souvent les jeunes enfants à travers le monde. Elle résulte généralement d'une alimentation inadéquate que d'un environnement malsain et des mauvaises conditions sanitaires [1]. La malnutrition par carence en micronutriments est celle survenant lors de la carence en micronutriments, le plus souvent secondaire à un déficit d'apport ou d'assimilation des micronutriments, elle est décrite par les Nations Unies comme la «faim invisible» ou «faim cachée» *(hidden hunger en anglais)* [1]. La carence en micronutriments représente une forme particulièrement dangereuse de malnutrition causée par la consommation insuffisante de vitamines et minéraux essentiels. L'OMS également la décrit comme la forme de malnutrition la plus grave compte tenu des rôles que jouent les oligoéléments dans le métabolisme humain [2]. Selon l'Unicef, dans les pays en développement, 30% de la population et presque un tiers des enfants sont sous-alimentés de façon chronique, soit un total de 777 millions d'individus; environ 11 millions d'enfants de moins de cinq ans meurent chaque année. Toujours selon l'Unicef, en 1998 plus de deux milliards de personnes souffrent de carence en micronutriments (soit un individu sur trois), et environ 39% d'enfants dans le monde souffrent de la carence en fer et sont donc anémiés [3]. L'éradication de la carence en fer est une priorité de santé publique et une des grandes causes sanitaires internationales. Les carences en micronutriments, aussi connues comme la faim cachée, demeurent un problème de santé publique important qui touche un tiers de la population mondiale [4]. Dans une étude publiée en 2005, l'anémie touchait plus de 47% des enfants de moins de 5 ans au niveau mondial [5]. Plusieurs études menées en Afrique francophone confirment l'étiologie de l'anémie mais affirment que 70 à 90% des jeunes enfants anémiés présentaient une carence martiale [6].

Les déficits en fer ont des retentissements hématologiques importants, mais aussi extra hématologiques, notamment sur les fonctions cognitives et sur l'immunité. Cette carence en fer reste la principale cause de l'anémie en Afrique. Elle a des effets néfastes sur la santé humaine, le développement socio-économique, avec une susceptibilité accrue aux infections, un risque élevé de mortalité maternelle et infantile, le développement cognitif et physique des enfants [7] ; elle altère donc leurs capacités d'apprentissage, leur insertion sociale et économique ultérieure [7, 8] et une plus faible productivité du travail des adultes ultérieure entrainant une altération de l'économie [7]. La faible biodisponibilité du fer explique, qu'en pathologie humaine, la carence martiale soit la plus répandue dans le monde, et constitue un véritable problème de santé publique pour certains pays [6]. Les anémies observées en Afrique francophone sont le plus souvent des anémies hypochromes microcytaires. Une étude réalisée au Sénégal confirme la sensibilité de la microcytose et de l'hypochromie comme critère de diagnostic d'une anémie ferriprive. L'anémie et la carence en fer du jeune enfant sont fortement prévalentes en Afrique francophone. Bien que le déficit d'apport en fer en soit un des facteurs essentiels, leurs causes sont multiples ; ce qui rend délicat non seulement le diagnostic, mais également le choix des stratégies de lutte à mettre en place [9]. Selon l'UNICEF, dans un rapport écrit par Mariane Flach, plus de 40% d'enfant de moins de cinq ans sont victimes de la malnutrition chronique en République Démocratique du Congo, soit le taux le plus élevé en Afrique centrale et de l'ouest. Les autres pays d'Afrique centrale les plus frappés sont la République Centrafricaine avec 40,7%, le Cameroun avec 32,5%, et le Congo Brazzaville avec 24,4%, selon le même rapport [1]. L'objectif de ce travail est de déterminer le taux de fer sérique et les facteurs liés à sa variation dans la malnutrition chez l'enfant vivant à Lubumbashi.

## Méthodes

Il s'agit d'une étude transversale faite d'une série de 182 enfants âgés de moins de 59 mois couvrant la période allant du 01 juillet 2013 au 31 décembre 2014. Ces enfants ont été recrutés parmi les enfants nouvellement admis au centre de réhabilitation ou de prise en charge de malnutrition aiguë sévère (MAS) de Lubumbashi et sur un échantillon fait dans la périphérie de la ville, au sein du village Kawama. Le diagnostic de malnutrition a été défini selon les critères de l'Organisation Mondiale de la Santé [10] à l'aide des indicateurs suivants: poids, taille et âge, permettant le calcul du z-score. L'état nutritionnel a été classifié de la manière suivante: état nutritionnel normal (Z-score supérieur ou égal à -1,00), malnutrition légère (Z-score compris entre -2,00 à -1,01), malnutrition modérée (Z-score compris entre -3,00 à -2,01) et malnutrition sévère (Z-score inférieur à -3,00). L'indice poids pour taille (WHZ) pour le diagnostic de la malnutrition aiguë, l'indice poids pour âge pour la malnutrition globale (WAZ), et l'indice taille pour âge pour la malnutrition chronique (HAZ). Le sexe, les signes cliniques à l'admission, une ponction veineuse effectuée au niveau des plis du coude et le sang gardé dans un tube puis centrifugé le même jour. Le sérum obtenu a été envoyé au laboratoire de l'Office Congolais de Contrôle (OCC) pour la préparation et la lecture à la spectrométrie d'absorption atomique ou à l'ICP de marque PERKIN-ELMER. Ainsi a été fait le dosage du fer dans le sérum. Les données ont été encodées et analysées avec le logiciel Epi Info vs 7,2, les Z-score ont été générés avec le logiciel Ena for smart et les références ont été traitées avec logiciel Zotero. Voici quelques définitions opérationnelles adoptées pour l'étude: plat familial; patte de farine de maïs ou de manioc + condiments (origine animale ou végétale), alimentation mixte; lait maternel + plat familial.

## Résultats

**Figure 1:**
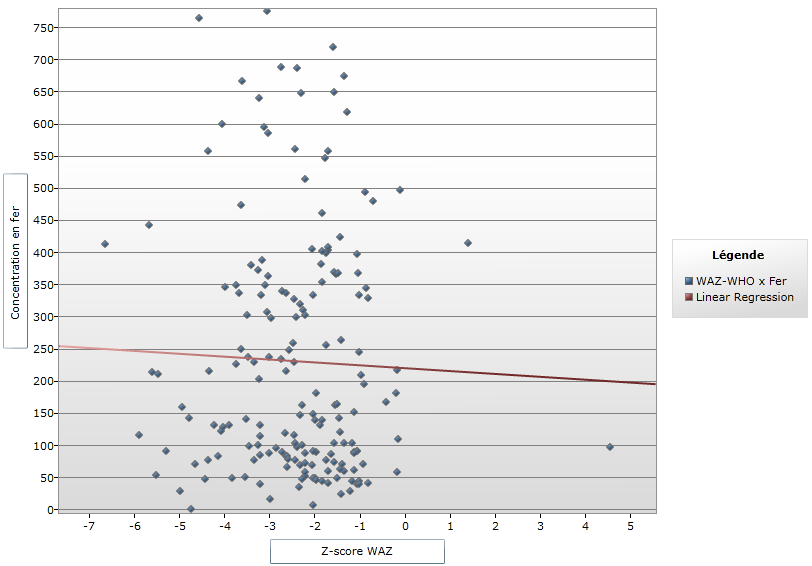
courbe de régression linéaire Z-score WAZ et concentration en fer

**Figure 2:**
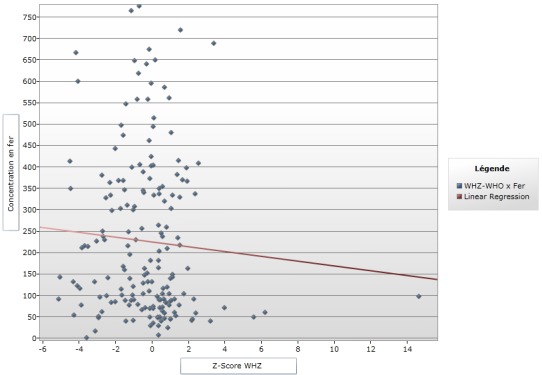
courbe de régression linéaire Z-score WHZ et concentration en fer

**Figure 3:**
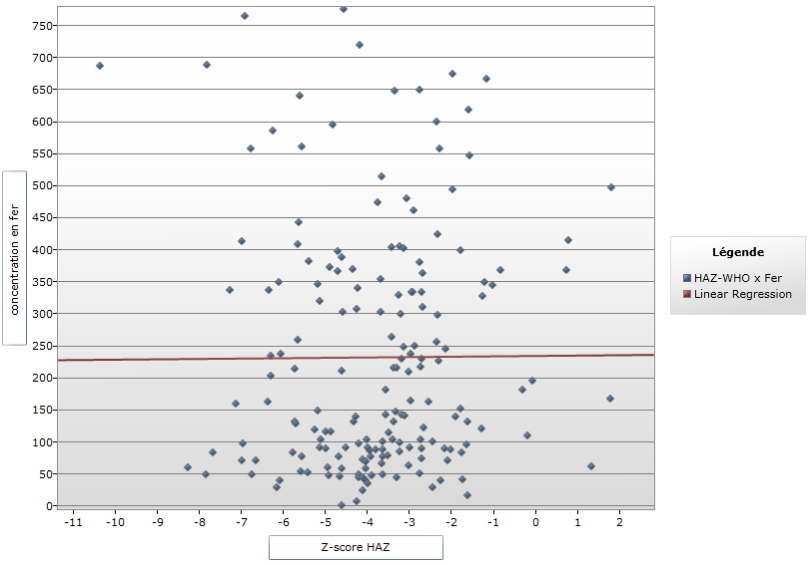
courbe de régression linéaire Z-score HAZ et concentration en fer

Le Tableau 1 montre que 170 enfants (93,4 %) ont une concentration sérique inférieure à la valeur normale par contre 12 enfant (6,59%) ont un taux de fer entre 610-1300 μg/L de fer sérique. La médiane était de 159,5 μg/L pour l'âge et le taux de fer, 170 (93,85) enfants avaient un taux <610 μg/L, dont 79 (47,02) qui avaient plus de 24 mois et 3 (1,78) âgés de moins de 6 mois. Pour les concentrations en fer situées entre 610-1300μg/L, 12 (6,15%) enfants avaient des taux normaux, dont 8 (72,72%) dans la tranche de 18-59 mois (Tableau 2). Pour ce qui est de la provenance, pour la ville de Lubumbashi, 108 (63,52%) enfants avaient un taux de fer <610 et 9 (75%) enfants avaient un taux de fer dans la tranche 610-1300μg/L alors que sur la population Kawama, 62 (36,47%) enfants avaient un taux de fer <610 Tableau 2). Concernant le régime alimentaire et le taux de fer <610 μg/L; 7 (4,11%) enfants qui étaient encore sous lait maternel exclusif parmi lesquelles d'autres avaient plus de 6 mois, et 122 (71,76%) enfants qui étaient sous le plat familial. Tandis que 12 enfants avaient le taux sérique entre 610-1300 μg/L dont 11 (91,66%) sous le plat familial (Tableau 2). Concernant le niveau d'étude de la mère et le taux en fer (Tableau 2); il ressort que sur 89 enfants pour qui les mères étaient de niveau secondaire, 82 (48,23%) enfants avaient un taux bas de fer (<610μg/L) par contre 7 (58,33%) enfants avaient une concentration sérique dans l'intervalle de 610-1300μg/L (normale). La diversification montre que 132 (88,00%) avaient une centration sérique en fer < 610 μg/L et 11 (7,69%) avaient une centration sérique en fer dans la fourchette de 610-1300 μg/L pour les enfants qui étaient diversifiés avant l'âge de 6 mois. Pour ceux qui ont été diversifiés après 6 mois 18 (22,00%) enfants avaient un taux sérique de fer < 610μg/L et 20 enfants n'avaient pas un âge bien déterminé. Ce tableau montre qu'il n'existe aucun lien statistiquement significatif entre le fer et les facteurs de risque où le p-value était < 0,05 sauf pour la variable provenance où le p est significatif. La Figure 1 montre que plus le Z-score augmente le taux en fer diminue. La forte proportion avait un Zscore entre -4' et -1 avec une médiane à -2,29 alors qu’une forte concentration du taux en fer se trouve en dessous de 150μg/L. Pour la courbe de régression linaire poids pour taille, il ressort que plus le Zscore augmente, le taux en fer diminue. La grande proportion de nos enfants se retrouve dans la tranche de zscore de -4 à 0 avec une médiane de -0,11 avec l'extrême à -6. Et avec une concentration en fer inférieure à 600μ/L soit autour de 250μ/ L et plus précisément inférieur à 150μ/L (Figure 2). La Figure 3 montre que la courbe de régression linéaire est parallèle à l'axe de z-score. Et le z-score étant autour de -2 et -6, avec une médiane à -3,65 avec une forte concentration en dessous de 200μg/L.

**Tableau 1:** Concentration en fer

Concentration en fer (μg/L)	Fréquence	Pourcentage
**<610**	170	93,41%
**610-1300**	12	6,59%
**Total**	182	100,00%

**Tableau 2:** Concentration en fer et paramètres sociodémographiques

Paramètres	Concentration en fer (μg/L)		
Tranche d'âge (années)	<610 (n=168)	610-1300(n=11)	p-value
**< 6**	3 (1,78)	0 (0,00)	0,72
**6-12**	17 (10,11)	1 (9,09)	
**12-18**	37 (22,02)	2 (18,18)	
**18-24**	32 (19,04)	4 (36,36)	
**> 24**	79 (47,02)	4 (36,36)	
**indéterminé**	2	1	
**Provenance**	**N=170**	**N=12**	0,42
**Lubumbashi**	108 (63,52)	9 (75,00)	
**Kawama**	62 (36,47)	3 (25,00)	
**Régime alimentaire**			0,50
**Alim mixte**	39 (22,94)	1 (8,33)	
**lait artificiel**	2 (1,17)	0 (0,00)	
**Lait maternel**	7 (4,11)	0 (0,00)	
**Plat familial**	122 (71,76)	11 (91,66)	
**Niveau d'étude de la mère**	**<610**	**610-1300**	0,76
**sans niveau**	29 (17,05)	2 (16,66)	
**Primaire**	59 (34,70)	3 (25,00)	
**Secondaire**	82 (48,23)	7 (58,33)	
**Age de diversification alimentaire**	**N=150**	**N=11**	0,22
**< 6**	132 (88,00)	11 (7,69)	
**> 6**	18 (22,00)	0 (0,00)	

## Discussion

En répartissant les données en fonction de la concentration sérique de fer nous avons constaté que 93,4% d'enfants malnutris vivent dans un état de carence martiale. Dans le même milieu, une étude similaire menée sur les éléments traces chez les enfants en état nutritionnel a rapporté de taux bas en fer sérique [11]. Ce résultat montre que notre population était plus carencée que celle de Berger qui, dans son étude sur les anémies par carence en fer en Thaïlande, avait trouvé que 60 % d'enfants avaient une carence en fer [12]. Cette étude nous permet de dire que la prévalence de la malnutrition par carence en fer est très élevée chez les enfants de moins de cinq ans à Lubumbashi avec un taux médian plus faible par rapport à la valeur observée chez les enfants malnutris sévère avec œdème de Kapolowe en 2006 par Shindano [4]. En 2005, Diouf et coll. ont rapporté que moins de la moitié des enfants consomment régulièrement des aliments riches en fer et seulement 12,6% d'entre eux reçoivent des suppléments de fer. La malnutrition (protéino-énergétique et vitaminique) contribue à la survenue d'une anémie. L'existence d'un statut inflammatoire, d'origine infectieuse ou parasitaire, modifie certains critères d'évaluation de la carence en fer dont le diagnostic devient alors délicat [6]. Concernant la répartition de la malnutrition en fonction de la tranche d'âge et la concentration en fer, il ressort que 79 (47,2%) enfants âgés de plus de 24 mois avaient une concentration sérique en fer inférieure à 610 μg/L de même que 89 (51,90%) enfants qui avaient un âge compris entre 1 et 24 mois. Concernant le niveau d'étude de la mère et le taux en fer; il ressort que sur 89 enfants malnutris leurs mères avaient un niveau secondaire, parmi ces enfants 82/89 (92,13%) avaient un taux bas de fer sérique (< 610μg/L) par contre 7/89 enfants (7,87%) avaient une concentration sérique en fer normale située dans l'intervalle de 610-1300μg/L. Contrairement à l'étude menée au Burkina Faso sur la malnutrition protéino-calorique et ses facteurs de risque, par Aouehougon qui a trouvé que 70,1% des mères des enfants malnutris n'avaient aucun niveau d'étude, 25,4% avaient un niveau primaire et seulement 4,1% des mères des enfants malnutris avaient un niveau post primaire. Il conclut que le niveau d'instruction de la mère est un facteur important déterminant dans le développement de la malnutrition des enfants [13]. Les études menées par l'Unicef en 1998 ont rapporté que les enfants des mères qui n'ont pas étudié ont trois fois plus le risque de développer la malnutrition avant cinq ans [14]. Ce qui est contraire à nos résultats où les mères avaient un bon niveau d'instruction soit un niveau secondaire et que leurs enfants ont plus présenté la malnutrition; ceci serait dû au fait soit qu'elles vont travailler et laissent les enfants à une tierce personne qui s'en occupe ou soit encore ceci serait lié à la pauvreté seulement et que ces mères ne sont pas à mesure de se procurer une alimentation riche en fer facilement assimilable.

Concernant le régime alimentaire et le taux de fer sérique <610 μg/L, notre étude reporte que 7 (100%) enfants qui étaient encore sous allaitement maternel exclusif, parmi eux se trouvait ceux qui avaient plus de 6 mois contre 122 (91,73%) enfants étaient sous le plat familial. Par rapport aux enfants malnutris qui avaient un taux sérique en fer normal, 12 (6,59%) enfants avaient été reportés avec un taux sérique entre 610-1300 μg/L dont 11 (8,27%) étaient nourris au plat familial. Une étude menée au Burkina a montré que 45,8% d'enfants malnutris étaient nourris au plat non varié type adulte mais malheureusement il n'a pas étudié la concentration en fer sérique dans son étude [13]. Ceci nous a amené à penser que l'introduction précoce d'une alimentation type adulte, très souvent non variée et le plus souvent pauvre en protéine d'origine animale et inadaptée aux besoins de l'enfant contribuerait à une carence en fer. Cette carence en fer qui empêcherait une bonne croissance de l'enfant et serait aussi un facteur déterminant dans le développement de la malnutrition des enfants. La diversification prématurée et inappropriée c'est-à-dire avant six mois d'âge, est un facteur non négligeable intervenant dans le développement de la malnutrition. La répartition des données en fonction de Z-score par rapport au poids pour l'âge et la concentration sérique de fer nous montre que la forte proportion des enfants se situait autour des Zscore entre -4 et -1 avec une médiane a -2, 29 avec une forte concentration du taux en fer se trouvent en dessous de 150μg/L. Ceci nous amène à dire que la carence en fer a influencé négativement sur la croissance pondérale des enfants. Nous n'avons pas non plus trouvé des littératures publiées par d'autres auteurs en rapport avec les explications de ce tableau. Selon les données dans le tableau concernant la répartition en fonction de Z-score par rapport à la taille pour l'âge, nous constatons que sur les 170 enfants ayant un déficit en fer, 54,7% avaient un Z-score Taille pour Age situé entre -3 et -2, tandis que 42,4% avaient un Z-score < -3, avec un total de 97,1%. En examinant la Figure 3, nous avons constaté que la courbe de régression logistique linéaire Z-score WAZ et concentration en fer seulement le Z-score PPA qui a une médiane à -2,29, ce résultat est presque le même que le Z-score moyen de Shindano à Kapolowe dans son groupe d'enfants malnutris avec œdèmes. Le Z-score TPA médian à -3,65, ce z-score est légèrement plus bas que celui de shindano à kapolowe (-2,7), mais pour le z-score PPT, nous avons enregistré un Z-score de -0,1 et Shindano à Kapolowe a eu un Z-score à -1,3. Nous pouvons dire que notre population est presque similaire à la population des malnutris de Shindano à kapolowe en 2012, qui était plus rurale [4].

## Conclusion

La carence en fer est la principale cause d'anémie dans la population infantile malnutrie de Lubumbashi et ses environs à leur admission dans une unité de prise en charge nutritionnelle pour malnutrition. Il ressort que 94,4% des enfants examinés ont présenté un taux sérique en fer inférieur à la valeur normale. La carence en fer a eu un impact négatif direct et remarquable sur la croissance des enfants examinés. Ce retard de croissance est remarquable sur le poids, mais surtout sur la taille qui était très inférieure à la moyenne. D'où la proposition des programmes de fortification en fer des différents aliments de consommation courante et/ou l'administration du fer à la première semaine de prise en charge de la malnutrition tout en traitant les complications (paludisme, les infections bactériennes et autres infections parasitaires).

### Etat des connaissances actuelles sur le sujet

- Le rôle de catalyseur joué par le fer dans la survenue des infections, a fait qu'il puisse être contre indiqué dans la prise en charge de la malnutrition;
- L'état de malnutrition constitue un terrain d'immuno-dépression d'où un risque élevé d'infection.

### Contribution de notre étude à la connaissance

- Il a été constaté au cours de cette étude la carence en fer observée chez les enfants malnutris et les enfants en bon état nutritionnel, d'où la nécessité de programmes de fortification en fer dans les différents aliments de consommation courante;
- L'administration du fer à la première semaine de prise en charge de la malnutrition tout en traitant les complications (paludisme, les infections bactériennes et autres infections parasitaires).

## Conflits d’intérêts

Les auteurs ne déclarent aucun conflit d’intérêts.
